# Surgical Antimicrobial Prophylaxis in Neonates and Children with Special High-Risk Conditions: A RAND/UCLA Appropriateness Method Consensus Study

**DOI:** 10.3390/antibiotics11020246

**Published:** 2022-02-14

**Authors:** Sonia Bianchini, Erika Rigotti, Laura Nicoletti, Sara Monaco, Cinzia Auriti, Elio Castagnola, Guido Castelli Gattinara, Maia De Luca, Luisa Galli, Silvia Garazzino, Stefania La Grutta, Laura Lancella, Andrea Lo Vecchio, Giuseppe Maglietta, Carlotta Montagnani, Nicola Petrosillo, Carlo Pietrasanta, Nicola Principi, Alessandra Simonini, Simonetta Tesoro, Elisabetta Venturini, Giorgio Piacentini, Mario Lima, Annamaria Staiano, Susanna Esposito, on behalf of the Peri-Operative Prophylaxis in Neonatal and Paediatric Age (POP-NeoPed) Study Group

**Affiliations:** 1 Pediatric Clinic, University Hospital, Department of Medicine and Surgery, University of Parma, 43126 Parma, Italy; bianchini.sonia@outlook.it (S.B.); laura.nicoletti@studenti.unipr.it (L.N.); s.monaco1410@gmail.com (S.M.); 2 Pediatric Unit, Department of Surgical Sciences, Dentistry, Gynecology and Pediatrics, University of Verona, 37124 Verona, Italy; erika.rigotti@aovr.veneto.it (E.R.); giorgio.piacentini@univr.it (G.P.); 3 Neonatology and Neonatal Intensive Care Unit, IRCCS Bambino Gesù Children’s Hospital, 00165 Rome, Italy; cinzia.auriti@opbg.net; 4 Infectious Diseases Unit, IRCCS Giannina Gaslini, 16147 Genoa, Italy; eliocastagnola@gaslini.org; 5 Unit of Immune and Infectious Diseases, IRCCS Bambino Gesu’ Children’s Hospital, 00165 Rome, Italy; guido.castelli@opbg.net (G.C.G.); maia.deluca@opbg.net (M.D.L.); alessandra.simonini@opbg.net (A.S.); 6 Pediatric Infectious Disease Unit, Meyer Children’s Hospital, 50139 Florence, Italy; luisa.galli@unifi.it (L.G.); carlotta.montagnani@meyer.it (C.M.); elisabetta.venturini@meyer.it (E.V.); 7 Paediatric Infectious Diseases Unit, Regina Margherita Children’s Hospital, University of Turin, 10122 Turin, Italy; silvia.garazzino@unito.it; 8 Institute for Biomedical Research and Innovation, National Research Council, 90146 Palermo, Italy; stefania.lagrutta@irib.cnr.it; 9 Paediatric and Infectious Disease Unit, Academic Department of Pediatrics, IRCCS Bambino Gesù Children’s Hospital, 00165 Rome, Italy; laura.lancella@opbg.net; 10 Department of Translational Medical Science, Section of Pediatrics, University of Naples Federico II, Via D. Montesano 49, 80131 Naples, Italy; andrealovecchio@gmail.com (A.L.V.); staiano@unina.it (A.S.); 11 Research and Innovation Unit, University Hospital of Parma, 43126 Parma, Italy; gmaglietta@ao.pr.it; 12 Infectious Disease and Infection Control Unit, Campus Bio-Medico, Medicine University Hospital, 00128 Rome, Italy; n.petrosillo@unicampus.it; 13 NICU, Fondazione IRCCS Ca’ Granda Ospedale Maggiore Policlinico, Department of Mother, Child and Infant, 20122 Milan, Italy; carlo.pietrasanta@unimi.it; 14 Università degli Studi di Milano, 20122 Milan, Italy; nicola.principi@unimi.it; 15 Division of Anesthesia, Analgesia, and Intensive Care, Department of Surgical and Biomedical Sciences, University of Perugia, 06129 Perugia, Italy; simonettatesoro@gmail.com; 16 Paediatric Surgery, IRCCS Azienda Ospedaliera-Universitaria di Bologna, 40138 Bologna, Italy; mario.lima@unibo.it

**Keywords:** antibiotic allergy, comorbidity, immunosuppression, MRSA, MSSA, multidrug resistant bacteria, splenectomy, surgical antibiotic prophylaxis

## Abstract

Surgical site infections (SSIs), which are a potential complications in surgical procedures, are associated with prolonged hospital stays and increased postoperative mortality rates, and they also have a significant economic impact on health systems. Data in literature regarding risk factors for SSIs in pediatric age are scarce, with consequent difficulties in the management of SSI prophylaxis and with antibiotic prescribing attitudes in the various surgical procedures that often tend to follow individual opinions. The lack of pediatric studies is even more evident when we consider surgeries performed in subjects with underlying conditions that may pose an increased risk of complications. In order to respond to this shortcoming, we developed a consensus document to define optimal surgical antimicrobial prophylaxis (SAP) in neonates and children with specific high-risk conditions. These included the following: (1) colonization by methicillin-resistant *Staphylococcus aureus* (MRSA) and by multidrug resistant (MDR) bacteria other than MRSA; (2) allergy to first-line antibiotics; (3) immunosuppression; (4) splenectomy; (5) comorbidity; (6) ongoing antibiotic therapy or prophylaxis; (7) coexisting infection at another site; (8) previous surgery in the last month; and (9) presurgery hospitalization lasting more than 2 weeks. This work, made possible by the multidisciplinary contribution of experts belonging to the most important Italian scientific societies, represents, in our opinion, the most up-to-date and comprehensive collection of recommendations relating to behaviors to be undertaken in a perioperative site in the presence of specific categories of patients at high-risk of complications during surgery. The application of uniform and shared protocols in these high-risk categories will improve surgical practice with a reduction in SSIs and consequent rationalization of resources and costs, as well as being able to limit the phenomenon of antimicrobial resistance.

## 1. Introduction

Surgical site infections (SSIs), which are a potential complication in surgical procedures, are associated with prolonged hospital stays and increased postoperative mortality rates, and they also have a significant economic impact on health systems [1,2]. It is estimated that about half of SSIs are avoidable by adequate preventive measures, of which perioperative antibiotics administration is one of the most effective in both adult and pediatric age [1,3,4].

SSI risk factors can be divided into non-modifiable intrinsic factors (i.e., related to the patient or the intervention) and modifiable extrinsic factors (i.e., related to the preoperative length of hospital stay). The main factors related to the patient are the presence of colonization with potentially pathogenic microorganisms, coexisting infections at another site, impaired immune response, high body mass index or poor nutritional status, underlying chronic disease, recent surgery, and presurgery prolonged hospitalization [5].

Most of the current knowledge on risk factors for SSIs has been obtained in adults, which explains why several Scientific Societies have been able to prepare precise guidelines for the prevention of the risk of developing SSIs [5]. Conversely, data in literature regarding risk factors for SSIs in pediatric age are scarce, with consequent difficulties in the management of SSIs prophylaxis and with antibiotic prescribing attitudes in the various surgical procedures that often tend to follow more individual opinions than schemes derived from the analysis of clinical studies conducted with scientifically flawless methods. The lack of pediatric studies is even more evident when we consider surgeries performed in subjects with underlying conditions that may pose an increased risk of complications. The real significance of this risk, the need for antibiotic prophylaxis, the type, and the effectiveness of the same remain in part or completely undetermined. In order to respond to this shortcoming, we developed a consensus document in order to define the optimal surgical antimicrobial prophylaxis (SAP) in neonates and children with specific high-risk conditions. These included the following: (1) colonization by methicillin-resistant *Staphylococcus aureus* (MRSA) and by multi-drug resistant (MDR) bacteria other than MRSA; (2) allergy to first-line antibiotics; (3) immunosuppression; (4) splenectomy; (5) comorbidity; (6) ongoing antibiotic therapy or prophylaxis; (7) coexisting infection at another site; (8) previous surgery in the last month; and (9) presurgery hospitalization lasting more than 2 weeks.

## 2. Methods

### 2.1. RAND/UCLA Appropriateness Method

This consensus document was realized using the Research and Development Corporation (RAND) and the University of California, Los Angeles (UCLA), appropriateness method. The RAND/UCLA method consists of the appropriateness evaluation of diagnostic and therapeutic procedures with suboptimal scientific evidence by a panel of experts [6]. According to the RAND method, a procedure is defined as “appropriate” if the expected benefits outweigh the expected negative consequences, with a wide margin that justifies it, regardless of the costs. In contrast, a procedure for which its expected risks outweigh the expected benefits is considered “inappropriate.” According to the RAND definition, experts who make an appropriateness/inappropriateness judgment must consider clinical benefits and not be influenced by economic considerations. Therefore, appropriateness is used in the evaluation of the risk/benefit ratio of a list of diagnostic, management, and therapeutic procedures [7].

### 2.2. Recruitment of Panelists

A multidisciplinary group of experts belonging to the main Italian scientific societies dealing with anti-infective therapy of children was selected. The following Scientific Societies were involved: Italian Society of Paediatrics (SIP); Italian Society of Neonatology (SIN); Italian Society of Paediatric Infectious Diseases (SITIP); Italian Society of Infectious and Tropical Diseases (SIMIT); Italian Society of Paediatric Surgery (SICP); Italian Society of Microbiology (SIM); Italian Society of Pharmacology (SIF); Italian Society of Anaesthesia and Neonatal and Paediatric Resuscitation (SARNEPI); and Italian Society of Childhood Respiratory Diseases (SIMRI). The panel of experts was made up of 52 medical doctors with at least a 5-year experience: paediatricians (*n* = 20), neonatologists (*n* = 6), infectious diseases specialists (*n* = 5), paediatric surgeons (*n* = 5), anesthetists (*n* = 8), pharmacologists (*n* = 5), and microbiologists (*n* = 3).

### 2.3. Generation of Scenarios

Initially, a literature search was performed with a selection of documents, including randomized studies, systematic reviews of the literature, meta-analyses, and guidelines on perioperative prophylaxis for the prevention of SSI during surgery in the presence of high-risk conditions. The literature search was carried out on the PubMed database, with a choice of articles in English published from 2000 to 2020. The key search terms were as follows: “antimicrobial prophylaxis” OR “antibiotic prophylaxis” AND “risk factor” OR “MRSA” or “methicillin-resistant *Staphylococcus aureus*” OR “multi-drug resistant bacteria” OR “MDR” OR “decolonization” OR “allergy” OR “immunosuppression” OR “immunodeficiency” OR “HIV” OR “splenectomy” OR “spleen” OR “underlying disease” OR “obesity” OR “malnutrition” OR “poor nutrition” OR “cardiopathy” OR “morbidity” OR “coexisting infection” OR “hospitalization” AND “neonate” OR “newborn” OR “paediatric” OR “pediatric” OR “children” OR “adolescent”. Subsequently, using the Patient/Problem/Population, Intervention, Comparison/Control/Comparator, and Outcome (PICO) model, a questionnaire was created on perioperative prophylaxis in specific high-risk conditions associated with increased infectious risks during surgery in neonatal and paediatric patients, which were divided into 13 clinical scenarios. Before administration, it was tested twice with a one-week interval to a convenience sample of 4 paediatricians, 2 neonatologists, 1 infectious disease specialist, 1 paediatric surgeon, 1 anesthetist, 1 pharmacologists, and 1 microbiologist. Then, 26 out of 52 experts were selected by the Scientific Societies for answering, and the questionnaire was administered to 11 paediatricians, 3 neonatologists, 2 infectious diseases specialists, 3 paediatric surgeons, 4 anesthetists, 2 pharmacologists, and 1 microbiologist.

### 2.4. Two-Round Consensus Process

On the basis of the scenarios, the questionnaire was submitted to experts on the “REDCap” online platform. Each question included the clinical scenario and possible answers were whether or not SAP was recommended for the scenario and, in case of its recommendation, a list with all antibiotics available on the EU market was provided so that the expert could select the antibiotics that he/she considered as first choice. The selected bibliographic material was made available to all panel members who were instructed on how to fill out the questionnaire. The experts answered anonymously to the questionnaire, and their judgement was expressed on a 1–9 scale, where “1” was considered definitely inappropriate, “5” was considered uncertain, and “9” was considered definitely appropriate. Intermediate values corresponded to different modulations of the judgement of inappropriateness (“2” and “3”), uncertainty (from “4” to “6”), and appropriateness (“7” and “8”). In evaluating each indication, each expert referred both to their own experience and clinical judgement and to the available scientific evidence. A free space was provided for any annotation or comment.

The first round of the questionnaire was blinded to other panel members. Multiple participation was not permitted by the platform, which also guaranteed the confidentiality and anonymity of the answers. The results of the survey were discussed in a collegial meeting with all 26 experts who answered the questionnaire to reach agreements and reduce eventual disagreements [7]. Clarifications, adaptations, and refinements of the indications and appropriateness ratings were made. A total of 13 recommendations were developed. Participants were asked to approve the recommendations in a second round during the following four weeks.

## 3. Results

### 3.1. Screening and Bacterial Colonization

The development of multiresistant bacteria to antimicrobial therapy (MDR), i.e., bacteria not susceptible to at least one agent in three or more classes of antimicrobial drugs [8], is an ever-growing phenomenon associated with high morbidity and mortality in both adults and children [9]. Among MDR bacteria implicated in SSIs, a central role is played by *S. aureus*, particularly in cases where the pathogen is resistant to methicillin (MRSA) [10]. In many cases, the antibiotic prophylaxis recommended for surgical interventions at risk of SSI is fully effective only against methicillin-sensitive *S. aureus* (MSSA), and the presence of MRSA can result in the development of serious diseases that are difficult to treat. In most cases, the development of *S. aureus* SSIs occurs in patients colonized by MRSA in the nose [10]. The screening of MRSA carriers and the decolonization is a safe and effective measure to reduce the risk of severe SSIs.

Other pathogens that can cause severe SSIs that cannot be prevented with SAP are *Acinetobacter* spp., *Pseudomonas aeruginosa* and various *Enterobacterales* (including *Klebsiella*, *Escherichia coli*, *Serratia*, and *Proteus*) [11].

#### 3.1.1. When to Perform Nasal Swab for *S. aureus*

**SCENARIO #1. Screening for MSSA and MRSA in a pediatric patient undergoing surgery**

Referring to the adult population, the most up-to-date studies with currently available data do not support universal screening for MSSA and MRSA in patients who are candidates for surgery, with the exception of cardiac and orthopedic surgery [5]. The studies included the analysis of the World Health Organization (WHO) guidelines performed on adult patients undergoing cardiac, orthopedic, gynecological, neurological, vascular, and gastrointestinal surgery, and the analysis came to a strong recommendation in favor of preoperative screening for *S. aureus* only for cardiothoracic and orthopedic interventions, while they recommended assessing the risk/benefit ratio for other types of surgery, based on the local epidemiology of MSSA and MRSA, as well as patient-related factors (i.e., previous *S. aureus* infection, carrier status known of MRSA, and known colonization status in body sites other than the nose) [12].

It is well known that adults undergoing cardiothoracic surgery are at increased risks for mediastinitis and other SSIs, which can be further complicated by MRSA [1]. Referring to the adult patient, Saraswat et al. stated that preoperative screening of *S. aureus* and decolonization has been associated with reduced colonization, transmission, and MRSA SSI in patients undergoing cardiac surgery [13]. Regarding the pediatric population, patients considered to be at high risks of MRSA infection are those with preoperative colonization, with a history of MRSA infection, subjects less than one month of age (i.e., neonates), and infants less than three months of age who have been hospitalized since birth or have a complex heart disorder [14]. With reference to orthopedic surgery of the adult patient, the prospective study conducted by Rao et al. suggested preoperative screening in patients who undergo orthopedic surgery (knee arthroplasty) and subsequent decolonization in order to reduce SSIs caused by *S. aureus* [15]. Regarding plastic surgery, Elward et al. stated that universal screening for MRSA is not approved, but that preoperative screening could be useful in some patient groups at increased risk of MRSA infection and in procedures involving implantable devices [16].

**Recommendation** **1.** *In the neonatal and pediatric patient who is undergoing ear-nose-throat (ENT), ophthalmology and abdominal, nephrourological, or plastic surgery in an emergency or elective regimen, the nasal routine screening for MSSA and MRSA detection is not recommended. In the patient undergoing neurosurgery or trans-nasal-sphenoid endoscopic surgery, in an emergency or elective regimen, although routine screening for S. aureus nasal colonization is not recommended, this may be strongly suggested in cases at high-risk of MRSA infection (i.e., those with MRSA preoperative colonization, those with a history of MRSA infection, neonates, and infants less than three months of age who have been hospitalized since birth or have a complex heart disorder). In patient who undergo to orthopedic or cardiothoracic surgery, in emergency or elective regimen, it performing nasal screening for colonization by Staphylococcus aureus is recommended.*

#### 3.1.2. When to Search for MDR Bacteria Other Than *S. aureus*

**SCENARIO #2. Screening for MDR other than MRSA in a pediatric patient undergoing surgery**

There are currently no indications in either the adult or pediatric population in favor of carrying out screening for colonization due to MDR bacteria. However, there is evidence for the effectiveness of early implementation of active surveillance (screening) by rectal screening for extended-spectrum beta-lactamase (ESBL) producing *Enterobacteriaceae* on admission to specific wards/units as neonatal intensive care unit (NICU) [17].

**Recommendation** **2.** *In the pediatric patient undergoing any type of surgery, in an emergency or elective regimen, routine screening is not recommended for the detection of colonization by MDR bacteria other than MRSA. In the neonatal patient admitted to NICU, rectal screening for ESBL producing Enterobacteriaceae detection is recommended.*

#### 3.1.3. Prophylaxis in Case of Colonization by MRSA or MDR

**SCENARIO #3. Perioperative prophylaxis in the patient colonized by MRSA**

Referring to the available clinical practice guidelines, in patients known to be colonized by MRSA who will undergo skin incision, it is useful to administer SAP with a single preoperative dose of vancomycin in addition to SAP already indicated for the type of surgery [5]. Given the lower efficacy of vancomycin compared to cefazolin in preventing MSSA-related SSIs, the combined use of cefazolin and vancomycin is considered appropriate in healthcare settings where both MRSA and MSSA infections are documented. Therefore, the use of vancomycin should not replace the SAP normally provided [5].

**Recommendation** **3.** *In the neonatal and pediatric patient colonized by MRSA who is undregoing any type of surgery, cefazolin at a dose of 30 mg/kg (maximum dose 2 g) IV combined with vancomycin at a dose of 15 mg/Kg (maximum dose 2 g) IV, both to be administered 30 min before surgery, is recommended.*

**SCENARIO #4. Perioperative prophylaxis in the patient colonized by MDRs other than MRSA**

There is currently no evidence in the literature in favor of specific perioperative antibiotic prophylaxis in the patient colonized by MDR bacteria other than MRSA. Only isolation and other measures to avoid the spread of the MDR bacteria are recommended [5]. However, rectal swab screening in elective abdominal surgery in settings with high prevalence of carbapenem-resistant *Enterobacteriaceae* could be considered in order to reduce hospital transmission of these pathogens [1].

**Recommendation** **4.** *In neonatal and pediatric patients colonized by MDR bacteria other than MRSA who undergo any type of surgery, the application of isolation and other measures to avoid the spread of the pathogen is recommended, but the routine execution of specific perioperative antibiotic prophylaxis is not recommended.*

#### 3.1.4. Decolonization

**SCENARIO #5. MRSA decolonization in the patient undergoing elective surgery**

In cases where it is deemed necessary to perform decolonization from MRSA, extensive systematic reviews and meta-analyses indicate performing decolonization with intranasal mupirocin ointment in the days preceding the surgery [12,17,18,19]. IDSA guidelines on perioperative prophylaxis provide, in addition to nasal mupirocin, a shower a day with soapy chlorhexidine (or povidone iodine for patients for whom chlorhexidine is contraindicated) for 5 days [5].

**Recommendation** **5.** *In the neonatal or pediatric patients colonized by MRSA who must undergo surgery, performing decolonization in the preoperative phase is recommended, using mupirocin nasal ointment at one application in each nostril three times a day and also a shower a day with soapy chlorhexidine (or povidone iodine for patients for whom chlorhexidine is contraindicated) for 5 days before surgery.*

**SCENARIO #6. Decolonization for MDR bacteria other than MRSA in the patient undergoing elective surgery**

There is currently no evidence in the literature in favor of decolonization of MDR bacteria other than MRSA in the preoperative phase.

**Recommendation** **6.** *In neonatal and pediatric patients colonized by MDR bacteria other than MRSA who undergo surgery, the routine execution of specific decolonization procedures in the preoperative phase is not recommended.*

### 3.2. Allergy to First-Line Antibiotics

**SCENARIO # 7. Perioperative prophylaxis in patients allergic to first-line antibiotics**

Beta-lactam antibiotics, especially cephalosporins, not only represent the main class of drugs used for the prevention of SSIs but also those most often involved in the determination of allergic reactions. In the presence of a suggestive clinical history or in case of documented allergy to β-lactams it is necessary, after confirming the existence of this problem, to define whether an alternative perioperative antibiotic prophylaxis is necessary and, in this case, which antibiotics to use. [20,21]. In patients allergic to penicillin, the risk of allergic symptoms after the administration of cephalosporins is usually limited to 1–10% of cases [22], with a maximum evidence for first generation cephalosporins. The third and fourth generation cephalosporins seem not to determine cross-reactivity phenomena [23]. Data collected in a retrospective study also demonstrated that the administration of cephalosporins to patients with skin testing positive for penicillin allergy or with a clinical history of anaphylaxis after the administration of this antibiotic never caused anaphylaxis [24].

Despite these data, in order to avoid risks, many authors recommend avoiding the administration of cephalosporins and carbapenems to subjects with clinical history or laboratory documentation of possible IgE-mediated allergy to penicillins who have suffered from severe clinical manifestations, anaphylaxis, and Steven–Johnson syndrome, including toxic epidermal necrolysis [5,25]. In these cases, second-line antibiotics generally recommended for the prevention of SSIs are vancomycin or clindamycin, although they have a narrower spectrum of action than first-generation cephalosporins, a less favorable pharmacokinetic profile, and some evidence of side effects [5,25]. Cephalosporins and carbapenems can, on the other hand, be administered to subjects with non-IgE mediated allergic reactions or nonlife-threatening reactions following the administration of penicillins [5,22,24]. At present, the data in the literature regarding the pediatric population are scarce, and the recommendations for this age group are extrapolated from what exists for the adult population.

**Recommendation** **7.** *In the neonatal or pediatric patient with strongly supposed or documented allergy to β-lactams undergoing surgery for which the perioperative administration of a cephalosporin is foreseen, the administration of vancomycin 15 mg/kg (dose maximum 2 g) IV or clindamycin 10 mg/kg (maximum dose 3 g) IV to be administered 30 min prior to surgery is recommended. In the event that the intervention involves the administration of a cephalosporin in association with other drugs, the latter will be used without any changes whatsoever in association.*

### 3.3. Immunodepression

**SCENARIO #8. Surgical prophylaxis in immunocompromised patients**

On a purely theoretical level, the presence of an immunodeficiency, be it congenital or acquired, should result in a greater risk of the onset of SSIs [26]. In reality, the increase in risk and the extent of this risk as a function of the type and severity of the immune deficiency on the one hand and the type of intervention on the other hand have not been studied, except in particular clinical conditions and in small numbers of adult patients. Hence, there is a difficulty in establishing precise and shared rules among experts for perioperative prophylaxis in case these patients have to undergo surgery. Some data are, however, available for adult patients with HIV infection, malignant diseases, or those undergoing transplantation for whom an increased risk of SSIs is documented compared to subjects without immune deficiency undergoing the same type of intervention [27,28,29,30,31,32]. However, even in these cases, it is not possible to establish precisely the type of SAP most suitable for the individual patient and whether this should be different from what is recommended for patients with normal immune system functions. In order to overcome this problem, some authors have been proposed prescribing SAP in every immunosuppressed patient undergoing surgery, even in cases where this is not normally expected, with the risk, however, of increasing the misuse of antibiotics without an effective practical advantage [5]. In any case, there are no ad hoc pediatric studies, and the procedures to be implemented in the child can only be derived from those used in the adult patient.

**Recommendation** **8.** *In the neonatal or pediatric patient with humoral or cell-mediated immune deficiency undergoing surgery, both in an emergency and elective regimen, perioperative antibiotic prophylaxis is recommended according to the indications provided for each single intervention for immunocompetent patients.*

### 3.4. Splenectomy

**SCENARIO #9. Splenectomy**

No data are available in the literature regarding perioperative antibiotic prophylaxis to be used specifically in the neonatal and pediatric populations undergoing splenectomy. Current clinical practice is based on recommendations based on studies carried out on adult patients. In the case of splenectomy performed in the elective regimen with a laparoscopic approach, the Lima and Russello groups recommend the administration of broad-spectrum antibiotic prophylaxis before surgery [33,34]. Samuk et al. reported the administration of a perioperative dose of antibiotic in all patients undergoing emergency splenectomy associated with a 24 h postoperative dose, underlining, however, the rarity of this type of intervention [35]. Current studies do not provide differential recommendations of perioperative antibiotic prophylaxis between laparoscopic and laparotomy approaches, indicating adequate perioperative administration of cefazolin [36].

**Recommendation** **9.** *In neonatal or pediatric patients, perioperative antibiotic prophylaxis with cefazolin at a dose of 30 mg/kg (maximum dose 2 g) IV within 30 min before surgery is recommended for splenectomy.*

### 3.5. Comorbidity

**SCENARIO #10. Perioperative antibiotic prophylaxis in patients with obesity or malnutrition**

Studies on the adult population have shown that obesity can be recognized as a risk factor for the development of SSIs, with a risk that seems to be directly related to body mass index [5]. From the pathophysiological point of view, there are no definitive conclusions, even if a study conducted in adults undergoing colorectal surgery has resulted in the hypothesis that the main factors that link the development of SSIs and the presence of obesity are the fact that obesity favors a decrease in oxygen tension in the wound and the reduced penetration of antibiotics into the tissues, as well as being associated with impaired immune function [37].

Regarding the importance of malnutrition as a risk factor for SSIs development, available data are very scarce. A study that evaluated the incidence of SSIs in a group of over 1000 adults with and without nutritional problems showed that the former had a higher risk of SSI (11.8% vs. 7.0%; *p* = 0.047) [38]. Furthermore, two meta-analyses of studies comparing the risk of SSI in adult patients with and without malnutrition when undergoing spinal surgery or orthopedic surgery confirmed the negative role of malnutrition [39].

There are no data available regarding perioperative prophylaxis in the obese or malnourished pediatric or adult patient. In the literature, there is evidence of a correlation between these conditions and the risk of SSI, but specific in-depth studies are lacking.

**Recommendation** **10.** *For the neonatal or pediatric patient suffering from obesity or malnutrition undergoing emergency or elective surgery, carrying out perioperative antibiotic prophylaxis using the drug(s) required for each specific intervention is recommended.*

**SCENARIO #11. Perioperative antibiotic prophylaxis in patients with comorbidities other than obesity or malnutrition**

In the literature, patients with comorbidities other than obesity and malnutrition at risk of complications during surgery are cardiopathic subjects, whether or not they have undergone previous operations. The available studies have been mainly addressed to consider the risk of developing endocarditis more than other infectious pathologies falling within SSI [40,41]. From the evaluation of several studies, it was concluded that patients with valve prostheses or prosthetic material used for valve repair; those who have already suffered from bacterial endocarditis; those with cyanogenic congenital heart disease (both not surgically corrected and corrected with the use of prostheses in the 6 months following the operation); and, finally, those undergoing heart transplantation who have developed valvulopathy are at greater risk of developing bacteremia and endocarditis [42]. Furthermore, although infectious complications have been described with all surgical procedures, it has been shown that operations on the mouth and teeth are clearly at greater risk than those involving the lower respiratory tract, the gastrointestinal tract and the genitourinary sector [42]. Finally, it has been established that, even among interventions involving the mouth and teeth, there are low-risk procedures including those involving local anesthesia of non-infected tissues, radiographs, positioning of orthodontic structures, extraction of teeth, and deciduous and dressings of lesions of the lips or oral mucosa following trauma [43]. All this has resulted in the suggestion that only subjects with high-risk cardiac alterations undergoing highly invasive oropharyngeal and dental surgeries should be subjected to SAP, while a specific prophylaxis was not strictly necessary for other forms of intervention [43].

**Recommendation** **11.** *Perioperative antibiotic prophylaxis in neonatal or pediatric patient with comorbidities other than obesity or malnutrition follows the same rules as patients without comorbidities. Exceptions are neonates or children with high-risk heart disease (i.e., those with valve prostheses or prosthetic material, those who have already suffered from bacterial endocarditis, those with cyanogenic congenital heart disease, and those undergoing heart transplantation who have developed valvulopathy) undergoing invasive oral, dental, or pharyngeal surgery. For these patients, the use of specific prophylaxis may be recommended. This may be based on the use of oral amoxicillin 50 mg/kg (maximum dose 2 g) or cefazolin 30 mg/kg (maximum dose 2 g) IV to be administered 30 min before surgery.*

### 3.6. Patients on Antibiotic Prophylaxis or Antibiotic Therapy or with Infection in Other Sites

**SCENARIO #12. Perioperative antibiotic prophylaxis in patient already undergoing antibiotic therapy or prophylaxis**

In situations in which the neonatal or pediatric patient who is a candidate for surgery is already undergoing antibiotic treatment for infection or is already undergoing antibiotic prophylaxis, the question arises as to which perioperative prophylaxis to set. In light of the scarce indications in the literature, it was decided to define the best behavior to be implemented in these circumstances on the basis of the indications present and those of the panel of experts. Currently, no clinical studies are available in the literature defining specific recommendations regarding perioperative antibiotic prophylaxis to be administered to patients who are already on antibiotic therapy or prophylaxis for other reasons or with infection in sites other than the surgical site. Bratzler et al. reported that patients receiving antimicrobial treatments for a remote infection prior to surgery should also receive SAP to ensure adequate serum and tissue levels of drugs with activity against probable pathogens involved in SSI for the duration of the operation [5]. If the agents used for therapy are also appropriate for SAP, it is sufficient to administer an extra dose 30 min before the surgical incision. If not, the recommended SAP for the planned procedure should be used according to standard recommendations. For patients with internal tubes or drains, the use of prophylactic antibiotics active against the pathogens potentially present in these devices may be considered prior to the procedure [5]. For chronic renal failure patients receiving vancomycin, a preoperative dose of cefazolin should be considered instead of an extra dose of vancomycin [5]. However, it should be remembered that, in most cases, these are patients on antibiotic prophylaxis or undergoing emergency surgery; in fact, elective surgery should always be postponed when the patient has an infection in another site [5].

**Recommendation** **12.** *In neonatal or pediatric patient already on antibiotic prophylaxis or already on antibiotic therapy for various reasons, or with coexisting infection in other sites (other than that in which surgery will take place) who undergo surgery, it is recommended to follow the indications provided for the single operation and to add prophylaxis with cefazolin at a dose of 30 mg/kg (maximum dose 2 g) IV to be administered 30 min before surgery if this is not already planned.*

### 3.7. Previous Intervention or Prolonged Hospitalization

**SCENARIO #13. Perioperative antibiotic prophylaxis in patients undergoing previous surgery or with prolonged hospitalization**

Patients who have recently undergone surgery or with prolonged hospitalization are considered to be at high risks of developing SSIs due to their greater frailty than the general population. Surgery and anesthesia result in a variety of metabolic and endocrine responses, resulting in a generalized state of immunosuppression in the postoperative period, the severity and duration of which can be determined in part by the extent of the initial surgical insult [44]. In all forms of surgery, perioperative immunosuppression can have immediate consequences for patients, including delayed wound healing, postoperative complications such as septic events, and slow postoperative recovery [45,46]. Furthermore, neonatal and pediatric patients have different physiology, postoperative metabolism, and needs than adults, which are conditions that must be considered in case of new surgery [47].

Length of preoperative hospital stay is considered another risk factor for SSIs. It has been reported that the risk of SSIs would increase 1.1-fold every 3 days of preoperative hospital stay [48]. Furthermore, the colonization of MDR bacteria during the hospital stay may be responsible for the increased risk of SSI [49,50].

There are currently limited data regarding specific surgical prophylaxis in recently operated or long-hospitalized children. Considering the risk of colonization by MDR pathogens in patients with prolonged hospitalization, screening for *S. aureus* (both MRSA and MSSA) is recommended before surgery by most experts [5]. This is valid also in patients with previous colonization or infections by MDR in order to confirm or exclude the presence of these difficult-to-treat bacteria. However, in cases of surgery in emergency, recommendations reported in scenarios #3, 4, and 12 should be followed.

**Recommendation** **13.** *In the neonatal or pediatric patient undergoing previous surgery in the last month and/or hospitalized for >2 weeks who underwent any type of surgery, it is recommended to carry out screening by nasal swab for the search for colonization by S. aureus (both MSSA and MRSA), and it is recommended to follow the indications for prophylaxis relating to the specific intervention.*

## 4. Discussion

SSIs are an ever-increasing phenomenon worldwide due to different factors and are responsible for generating important costs [51,52]. This cost depends on many factors, including the patient himself and the type of surgery [53]. There are few or no specific recommendations for perioperative prophylaxis in pediatric patients with high risks of complications during surgery. Since the need for indications on this topic is strong in those who provide daily care for surgical patients in neonatal and pediatric age, we have carried out this work, which is the result of the collaboration of a multidisciplinary group of experts.

Regarding the screening of multiresistant pathogens, the panel of experts concluded that screening for MRSA in cases of cardiothoracic and orthopedic surgery should be performed, in line with what was stated in the literature relating to adult patients, and in the case of prolonged hospitalization [13,15]. In addition, screening for MRSA has been indicated as possible in patients who are candidates for neurosurgery or transnasal-sphenoid endoscopic surgery, where there is no indication to perform routine research but where this can be considered on a case-by-case basis. For decolonization, although there is general agreement on the use of nasal mupirocin [16], there are no univocal indications on timing and dosages: Our panel of experts has indicated an application in each nostril three times a day for 5 days before the surgery as optimal. In addition, our panel recommended also a shower a day with soapy chlorhexidine (or povidone iodine for patients in whom chlorhexidine is contraindicated) for 5 days before surgery, as already recommended by other experts in adult patients [5]. In patients known to be colonized by MRSA, the panel concluded that vancomycin is added to perioperative prophylaxis, in line with existing guidelines [5].

In light of the emergence of other multi-resistant pathogens, it was the objective of this study to provide indications on the need for preoperative screening and on any modifications of perioperative prophylaxis in patients known as carriers. On this point, the panel of experts concluded that there is no need for eradication treatment, with an indication in any case to the rigorous application of infection surveillance, especially in high-risk units/wards (i.e., NICU) and of containment measures in any unit/ward when dealing with a carrier patient. In cases of neonates, this is valid for those admitted to NICU after birth and those admitted for surgical interventions. Furthermore, a different perioperative prophylaxis has not been indicated in patients with MDR germs.

In patients allergic to beta-lactams, vancomycin or clindamycin are considered as alternatives when SAP is recommended, although first-line cephalosporins may be administered to subjects with non-IgE mediated allergic reactions or non-life-threatening reactions following the administration of penicillin [5,22,24].

In the perioperative prophylaxis of the immunocompromised patient, the incorrect use of antibiotics represents a concrete risk, both for the characteristics of the patient and for the lack of shared guidelines. The panel of experts defined the absence of a need for a different perioperative prophylaxis in this category of patients, avoiding the risk of antibiotic abuse. On the contrary, in the case of splenectomy, although there are no clear recommendations, our panel of experts agree on the use of cefazolin presurgery, avoiding the administration of other broad-spectrum antibiotics.

With respect to regards patient with obesity, the poverty of data and evidence in the pediatric field emerged. The literature reports that obesity is associated with a higher risk of SSIs in the adult population, but at the same time, no molecules or dosages other than those indicated for the normal weight patient are indicated for antibiotic prophylaxis [50]. In accordance with this line, the panel of experts concluded that there is no need to perform specific perioperative prophylaxis in an obese pediatric patient. Given the global increase in obesity and its high prevalence in pediatric age, specific studies on children would make it possible to verify the incidence of SSIs in this population and, if necessary, to clarify the pathophysiological mechanism so as to be able to refine strategies to reduce their incidence. In fact, it should be considered that the observations made to date in the literature refer only to the adult population, but there are important differences between obese children and adults, which could also determine a different risk for pediatric subjects.

Moreover, with regard to malnutrition, there are currently no indications for perioperative prophylaxis. The reason for this is probably linked to the location of most cases of malnutrition in low-resource countries, where fewer clinical trials are conducted, with isolated cases in high-development countries, where complex pictures associated with serious comorbidities are sometimes observed. The present work felt the need to answer this question and the panel of experts defined the absence of the need for a different perioperative prophylaxis in this category of subjects. Moreover, in this case, it remains to be clarified with new studies whether malnutrition is associated with a higher incidence of SSIs and what are the physiopathological mechanisms underlying the phenomenon.

A specific question arose about the pediatric patient who, at the time of surgery, is already on prophylaxis or on antibiotic treatment. It is, in fact, a situation frequently encountered in pediatric ages, which can reasonably result in doubts in the management of perioperative prophylaxis in the presence of scarce indications in the literature. Therefore, the position indicated by the panel of experts is of great interest, which recommends following the indications provided for the single intervention and for the specific patient and to add prophylaxis with cefazolin if this is not foreseen. Similarly, for patients who have recently undergone surgery or have been hospitalized for a long time, experts recommend following the directions provided for each individual procedure, although in these cases, carrying out screening by nasal swab for the search for colonization by *S. aureus* is recommended (both MSSA and MRSA).

This work aimed to respond to issues that are still little addressed, with the ambition to fill current shortcomings. The definition of specific categories of patients with risk factors was formulated from scratch on the basis of knowledge of the critical points on perioperative prophylaxis of children and newborns. The specific scenarios developed are intended to guide the healthcare professional in practice so as to ensure a better and standardized management of the neonatal and pediatric patient. The strengths of the work are an updated literature review, the use of a rigorous analysis method (RAND/UCLA), the involvement of a large number of exponents of the most important Italian scientific societies, and the specific consideration of age. The potential limitation of this study is the scarcity of data in the literature, which is partly overcome by the involvement of numerous and selected experts. On the other hand, the lack of pediatric studies on the selected topics did not permit the use of GRADE methodology, and the complexity of the topics required an online face-to-face meeting with all participants.

## 5. Conclusions

This study, made possible by the multidisciplinary contribution of experts belonging to the most important Italian scientific societies, represents, in our opinion, the most up-to-date and comprehensive collection of recommendations relating to the behaviors to be undertaken in a perioperative site in the presence of specific categories of patients at high risk of complications during surgery. Table 1 summarizes the recommendations. The application of uniform and shared protocols in these high-risk categories will improve surgical practice, with a reduction in SSIs and consequent rationalization of resources and costs, as well as being able to limit the phenomenon of antimicrobial resistance. As soon as our consensus document is implemented by the Italian Scientific Societies, it will be interesting to analyze its clinical and economic impact in our geographical context. However, our recommendations could be generalized also in low- and middle-income countries where the impacts of simple, cost-effective, sustainable, and adaptable strategies on the reduction in morbidity risk and the associated costs have been recently highlighted [54,55].

## Figures and Tables

**Table 1:** Surgical antimicrobial prophylaxis (SAP) for neonates and children with special high-risk conditions.

Clinical Scenario	Recommendation
Screening for MSSA and MRSA	In neonatal or pediatric patients who is uundergoing ENT, ophthalmology, abdominal, nephro-urological, or plastic surgery, in an emergency or elective regimen, nasal routine screening for MSSA and MRSA detection is not recommended. In the patient undergoing neurosurgery or transnasal-sphenoid endoscopic surgery, in an emergency or elective regimen, although routine screening for *S. aureus* nasal colonization is not recommended, this may be strongly suggested in cases at high risk of MRSA infection (i.e., those with MRSA preoperative colonization, those with a history of MRSA infection, neonates, and infants less than three months of age who have been hospitalized since birth or have a complex heart disorder). In patients who undergo orthopedic or cardiothoracic surgery in emergency or elective regimen, performing nasal screening for colonization by *Staphylococcus aureus* is recommended.
Screening for MDR other than MRSA	In the pediatric patient undergoing any type of surgery, in an emergency or elective regimen, routine screening is not recommended for the detection of colonization by MDR bacteria other than MRSA. In the neonatal patient admitted to NICU, rectal screening for ESBL producing *Enterobacteriaceae* detection is recommended.
Patient colonized by MRSA	In the neonatal or pediatric patients colonized by MRSA who undergo any type of surgery, cefazolin at a dose of 30 mg/kg (maximum dose 2 g) IV combined with vancomycin at a dose of 15 mg/Kg (maximum dose 2 g) IV, both to be administered 30 min before surgery, is recommended.
Patient colonized by MDR bacteria other than MRSA	In neonatal or pediatric patients colonized by MDR bacteria other than MRSA who undergo any type of surgery, the application of isolation and other measures to avoid the spread of the pathogen is recommended, but the routine execution of specific perioperative antibiotic prophylaxis is not recommended.
MRSA decolonization	In the neonatal or pediatric patient colonized by MRSA who must undergo surgery, it is recommended to perform decolonization in the preoperative phase, using mupirocin nasal ointment one application in each nostril 3 times a day and also a shower a day with soapy chlorhexidine (or povidone iodine for patients in which chlorhexidine is contraindicated) for 5 days before surgery.
Decolonization for MDR bacteria other than MRSA	In neonatal or pediatric patients colonized by MDR bacteria other than MRSA who undergo surgery, the routine execution of specific decolonization procedures in the preoperative phase is not recommended.
Patients allergic to first-line antibiotics	In the neonatal or pediatric patient with strongly supposed or documented allergy to β-lactams undergoing surgery for which the perioperative administration of a cephalosporin is foreseen, the administration of vancomycin 15 mg/kg (dose maximum 2 g) IV or clindamycin 10 mg/kg (maximum dose 3 g) IV to be administered 30 min prior to surgery is recommended. In the event that intervention involves the administration of a cephalosporin in association with other drugs, the latter will be used without any changes whatsoever in association.
Immunocompromised patients	In the neonatal or pediatric patient with humoral or cell-mediated immune deficiency undergoing surgery, both in an emergency and elective regimen, perioperative antibiotic prophylaxis is recommended according to the indications provided for each single intervention for immunocompetent patients.
Splenectomy	In neonatal or pediatric patients, perioperative antibiotic prophylaxis with cefazolin at a dose of 30 mg/kg (maximum dose 2 g) IV within 30 min before surgery is recommended for splenectomy.
Patients with obesity or malnutrition	For neonatal or pediatric patients suffering from obesity or malnutrition who undergo emergency or elective surgery, it is recommended to carry out perioperative antibiotic prophylaxis using the drug (s) required for each specific intervention.
Patients with comorbidities other than obesity or malnutrition	Perioperative antibiotic prophylaxis in neonatal or pediatric patient with comorbidities other than obesity or malnutrition follows the same rules as for patients without comorbidities. Exceptions are neonates or children with high-risk heart disease (i.e., those with valve prostheses or prosthetic material, those who have already suffered from bacterial endocarditis, those with cyanogenic congenital heart disease, and those undergoing heart transplantation who have developed valvulopathy) undergoing invasive oral, dental, or pharyngeal surgery. For these patients, the use of specific prophylaxis may be recommended. This may be based on the use of oral amoxicillin 50 mg/kg (maximum dose 2 g) or cefazolin 30 mg/kg (maximum dose 2 g) IV to be administered 30 min before surgery.
Perioperative antibiotic prophylaxis in patients already undergoing antibiotic therapy or prophylaxis	In neonatal or pediatric patient already on antibiotic prophylaxis or already on antibiotic therapy for various reasons, or with coexisting infection in other sites (other than that in which surgery will take place) who undergo surgery, it is recommended to follow the indications provided for the single operation and to add prophylaxis with cefazolin at a dose of 30 mg/kg (maximum dose 2 g) IV to be administered 30 min before surgery if this is not already planned.
Patients undergoing previous surgery or with prolonged hospitalization	In the neonatal or pediatric patient undergoing previous surgery in the last month and/or hospitalized for >2 weeks who is undergoing any type of surgery, it is recommended to carry out screening by nasal swab for the search for colonization by S. aureus (both MSSA and MRSA), and it is recommended to follow the indications for prophylaxis relating to the specific intervention.

ENT, ear-nose-throat; ESBL, extended-spectrum beta-lactamase; MDR, multidrug resistant; MRSA, methicillin-resistant *Staphylococcus aureus*; MSSA, methicillin-susceptible *Staphylococcus aureus*; NICU, neonatal intensive care unit.

## Data Availability

All the data are included in the manuscript.
